# Rehabilitation after paediatric acquired brain injury: Longitudinal change in content and effect on recovery

**DOI:** 10.1111/dmcn.15199

**Published:** 2022-03-09

**Authors:** Rob J. Forsyth, Liz Roberts, Rob Henderson, Lorna Wales

**Affiliations:** ^1^ Translational and Clinical Research Institute Newcastle University Newcastle upon Tyne UK; ^2^ Newcastle upon Tyne Hospitals NHS Foundation Trust Newcastle upon Tyne UK; ^3^ The Children’s Trust Harrison Research Centre Tadworth UK; ^4^ School of Mathematics, Statistics and Physics Newcastle University Newcastle upon Tyne UK

## Abstract

**Aim:**

To describe cross‐sectional and longitudinal variation in neurorehabilitation content provided to young people after severe paediatric acquired brain injury (pABI) and to relate this to observed functional recovery.

**Method:**

This was an observational study in a cohort of admissions to a residential neurorehabilitation centre. Recovery was described using the Pediatric Evaluation of Disability – Computer Adaptive Testing instrument. Rehabilitation content was measured using the recently described Paediatric Rehabilitation Ingredients Measure (PRISM) and examined using multidimensional scaling.

**Results:**

The PRISM reveals wide variation in rehabilitation content between and during admissions primarily reflecting proportions of child active practice, child emotional support, and other management of body structure and function. Rehabilitation content is predicted by pre‐admission recovery, suggesting therapist decisions in designing rehabilitation programmes are shaped by their initial expectations of recovery. However, significant correlations persist between plausibly‐related aspects of delivered therapy and observed post‐admission recovery after adjusting for such effects.

**Interpretation:**

The PRISM approach to the analysis of rehabilitation content shows promise in that it demonstrates significant correlations between plausibly‐related aspects of delivered therapy and observed recovery that have been hard to identify with other approaches. However, rigorous, causal analysis will be required to truly understand the contributions of rehabilitation to recovery after pABI.

**What this paper adds:**

- Rehabilitation content varies widely between, and during, admissions for neurorehabilitation after paediatric acquire brain injury.
- Strong correlations are seen between plausibly‐related aspects of rehabilitation content and observed recovery, though careful interpretation is necessary.

## What this paper adds

We examine the content of neurorehabilitation provided to young people recovering after severe paediatric acquired brain injury (pABI). Content varies widely between and during admissions and is predicted by pre‐admission recovery suggesting rehabilitation programmes are shaped by therapists' expectations of recovery. However significant correlations persist between plausibly‐related aspects of delivered therapy and observed post‐admission recovery after adjusting for such effects. Rigorous causal analysis will be required to truly understand the contributions of rehabilitation to recovery after pABI

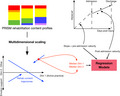

This original article is commented on by Whyte on pages 1057–1058 of this issue.

**Abbreviations**
MDS: Multidimensional scaling
pABI: Paediatric acquired brain injury
PEDI‐CAT: Pediatric Evaluation of Disability – Computer Adaptive Testing
PRISM: Paediatric Rehabilitation Ingredients Measure

Paediatric acquired brain injury (pABI) represents an increasing health challenge as developments in intensive care medicine have reduced the rate of mortality from critical illnesses.^1^ Rehabilitation remains the mainstay of the clinical response, however, its contribution to outcome, and whether and how this can be improved, remains poorly understood.^2^

Achieving a better understanding of the role of rehabilitation in improving recovery after pABI presents three distinct challenges.^3^ First, models of severity‐adjusted outcome after pABI are needed.^4^, ^5^, ^6^ The residuals of these models (i.e. the variation in outcome not explained by initial severity and related predictors) are where possible rehabilitation treatment effects lie.^7^, ^8^ Second, any such variances in outcome need to be related to the rehabilitation received, which in turn requires methods for quantification of the ‘ingredients’ and ‘dosage’ of the rehabilitation delivered. The third challenge is to acknowledge that therapists adjust rehabilitation treatment content in response to the recovery they are seeing; hence, there may be a reciprocal relationship between treatment choices and ongoing recovery that complicates analysis.

This paper focuses on the second and third of these challenges. We have recently described a Paediatric Rehabilitation Ingredients Measure (PRISM) as a practical means of describing rehabilitation content.^9^ The motivation for the development of PRISM was recognition that basic measures of rehabilitation dose such as ‘therapist contact hours’ are meaningless if the aims and content of that rehabilitation—and, thus, what might plausibly be expected to change as a result—are unspecified. For example, although the total rehabilitation dose for a child with a severe disorder of consciousness (with very limited awareness of their surroundings) will typically be very high, PRISM confirms that the proportion of the effort towards active practice and relearning of movement and skill is low.^9^ The team’s focus is elsewhere: on prevention of deformity, ensuring comfort and tolerance of care procedures, enabling participation through environmental adaptation, and family support. Therefore progress might be expected in domains such as family competence and emotional health, but not functional gain. Throughout this paper we refer to pairings of rehabilitation content (as reflected by PRISM) and aspects of outcome that can be expected to change as a result of that rehabilitation, as ‘plausibly‐related’ (i.e. there is a strong a priori case, based on treatment theory, that the former should effect improvements in the latter). One such pairing that is highly salient to families^10^ in the early post‐acute rehabilitation phase is the recovery of gross motor function (standing, walking) and ‘active practice of skills’ (one of the available PRISM content domains). We have recently reported pilot findings of relationships between the PRISM‐derived proportion of active practice and gross motor recovery trajectories as assessed by the Gross Motor Function Measure.^3^ Although strong positive correlations were noted, it also seemed likely (with reference to the third challenge mentioned above) that the decision to include a high proportion of active practice in rehabilitation was as much a response to, than a cause of, observed faster recovery of gross motor function.^3^

This paper aimed to describe variations in rehabilitation content during and between different individuals’ admissions, and to examine relationships between recovery to date, rehabilitation treatment content, and ongoing recovery. Compared to our previous work,^3^ it includes a broader range of both rehabilitation treatment ‘ingredients’ (not just active practice) and aspects of outcome (not just gross motor function) in a larger cohort.

## METHOD

### Participants

The children in this study received residential neurorehabilitation at The Children’s Trust, Tadworth, Surrey between June 2019 and October 2020.

### PRISM

The development of PRISM is described elsewhere^9^ and online (https://www.youtube.com/watch?v=QW6ZwY1gacs and https://www.youtube.com/watch?v=‐GSjSiM_apE). It is used to describe the areas of focus of the rehabilitation multidisciplinary team, expressed as proportions among a range of required items selected from a pre‐defined ‘menu’ of 11 available options (active practice of skills by child; active practice by family of newly‐required skills; imparting knowledge and understanding to child; imparting knowledge and understanding to family; imparting knowledge and understanding to other professionals; other management of child’s body structure and function; emotional support of child; emotional support of family; adaptation of home environment; adaptation of community environment; advocacy for child with other professionals). It uses the analytical hierarchy process^11^ to assist with these estimations. The practicalities of the analytical hierarchy process encourage restraint in the number of items considered pertinent at any one time, and in practice it is rarely necessary to give more than four or five items non‐zero scores. The components of a PRISM score sum to 1; if, for example, the proportion of rehabilitation effort allocated to active practice is estimated at 0.8, the sum of all other fractions is limited to 0.2. PRISM data require compositional data‐analysis methods that acknowledge this interdependency.

PRISM scores were estimated at monthly multidisciplinary team meetings using a bespoke online analytical hierarchy process calculator. PRISM scores come with an internal consistency ratio: a ratio < 10% is deemed acceptable and all PRISM estimations met this criterion.

### Pediatric Evaluation of Disability Inventory – Computer Adaptive Testing

The functional outcome assessment tool used in these analyses was the Pediatric Evaluation of Disability Inventory – Computer Adaptive Testing (PEDI‐CAT),^12^, ^13^ an adaptation of the original Pediatric Inventory of Disability Inventory with robust interval (‘Rasch’) scale properties. PEDI‐CAT ratings were administered using the Apple version of the software (version 1.4.0).^13^ PEDI‐CAT scores are provided in four domains: Daily Activity, Mobility, Social/Cognitive, and Responsibility and are available as raw scaled scores and age‐adjusted normed scores (as typical expectations of function increase with age). However, the latter are only provided for whole‐year chronological age‐bands. This meant that a child who happened to have a birthday during rehabilitation would fall abruptly on normed scores on that day, thus, raw scaled scores were used with age as a covariate where appropriate. The PEDI‐CAT tool allows two assessment types: ‘speedy’ and ‘content‐balanced’. The former was used throughout the study.

PEDI‐CAT scores were obtained at admission and discharge. PEDI‐CAT velocities (rates of change) were used rather than absolute score changes between admission and discharge. This was to avoid the potentially confounding effect of length of stay that varies between children and is itself a proxy for injury severity.^14^, ^15^ Two velocities were calculated for each of the four domains (Daily Activity, Mobility, Social/Cognitive, and Responsibility): one for the preadmission period (i.e. admission PEDI‐CAT score divided by the number of days between injury and admission), and one for the admission period (the slope of a best fit line through admission, discharge, and any intermediate assessments). To ensure that recovery was assessed over the same period that PRISM‐capture of rehabilitation content was available, cases where the admission PEDI‐CAT assessment preceded the first PRISM assessment by more than 45 days were omitted. Likewise, individual PEDI‐CAT observations made more than 45 days after the last PRISM assessment were ignored in calculating post‐admission PEDI‐CAT velocity. The pre‐admission velocity calculation assumes a minimum possible PEDI‐CAT score on the day of injury; because the PEDI‐CAT is normed to a 20 to 80 scale, a score of 20 on the day of injury was assumed.

The PEDI‐CAT ability estimate algorithm generates a (mis)fit score for each domain that if large (>2.0) suggests an unexpected pattern of scores across items and that the overall scaled score should be interpreted with caution; there were no instances of this in the data set.

As an observational study that used routinely collected clinical data, with no consequences for or effects on patient care, this study was deemed a service evaluation by standard criteria (www.hra‐decisiontools.org.uk), therefore a research ethics review was not sought. The study was endorsed by The Children’s Trust research committee (TCT049 May 2017).

### Statistical analysis

Statistical analyses were performed in R version 3.6.2 (R Foundation for Statistical Computing, Vienna, Austria). Multiple approaches to the visualization of PRISM data were used: hierarchical clustering was examined using *pvclust*; principal components analysis was performed using the *princomp* function and multidimensional scaling (MDS) using *cmdscale* (Appendix S1). MDS is a means of visualizing similarity in a data set: PRISM observations are converted to points on an *x, y* scatterplot, arranged so that the distances between them reflect the dissimilarities in their full PRISM scores as accurately as possible. The *rcomp* function from the *compositional* R package was used to transform PRISM data before further analysis in light of its compositional nature.

## RESULTS

PRISM data were available for 61 children (53% male), of a total of 94 admissions during this period. Median age at injury (range; interquartile range [IQR]) was 11 years 2 months (11 months–17 years 4 months; 4 years–14 years 7 months); interval from injury to admission 128 days (13–3854; 71–286). Injury mechanism was trauma (*n* = 15), anoxia (*n* = 9), and other (predominantly various types of stroke and tumour) (*n* = 37). There were no statistically significant differences in sex, age at injury, interval from injury to admission, or injury mechanism between the 61 children with PRISM scores and the 33 without. PEDI‐CAT data were available for 51 out of 61 children.

### PRISM

**FIGURE 1:**
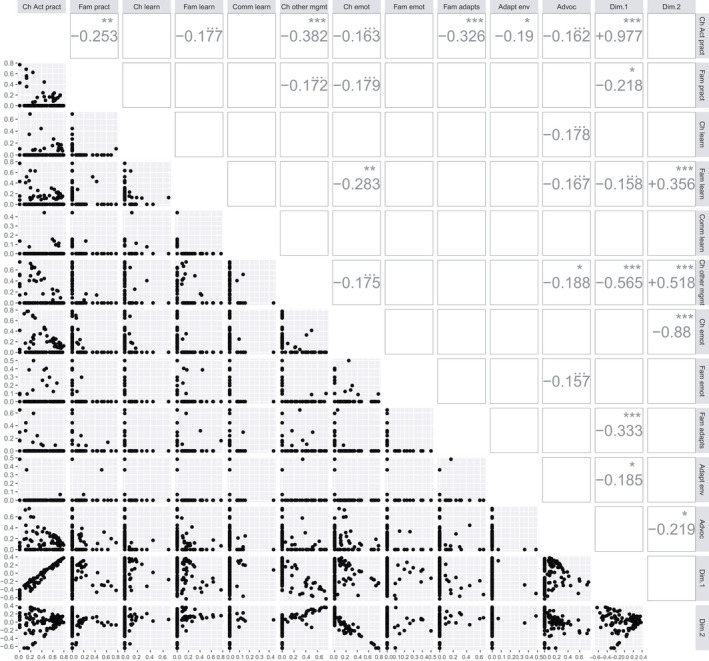
Pairwise scatterplots (lower left) and corresponding Pearson correlations (upper right) of the Paediatric Rehabilitation Ingredients Measure (PRISM) domain scores together with the Dimension 1 and Dimension 2 coordinates of the two‐dimensional multidimensional scaling. For clarity, Pearson correlations are only shown where the *p* value is < 0.1. Statistical significance: •, *p* < 0.1; *, *p* < 0.05; **, *p* < 0.01; ***, *p* < 0.001. Abbreviations: Cld, child; Fam, family; Comm, community; pract, active practice of skills; learn, learning of explicit knowledge; emot, promoting emotional health; other, other management of body structure and function; supp, supporting family through provision of aids and adaptions; adapt env, adapting physical community environment outside home; advoc, advocating for child and family in community

One hundred and eighteen PRISM observations were made in 61 children (median 2 observations per child, IQR 1–2) at approximately monthly intervals. Figure 1 shows pairwise scatterplots of the PRISM proportions. ‘L‐shaped’ distributions (with all points near the *x* = 0 or *y* = 0 axes) were common, implying that many possible ingredient pairs were treated as mutually exclusive. If any one ingredient was scored, none of the others were. Modest but highly significant negative correlations were evident between the child, active practice, and each of family practice, child, other management, family adaptations, and environmental adaptation (Figure 1). These observations suggested that it would be appropriate to apply dimensionality‐reduction approaches to PRISM data.

### Dimensionality reduction

**FIGURE 2:**
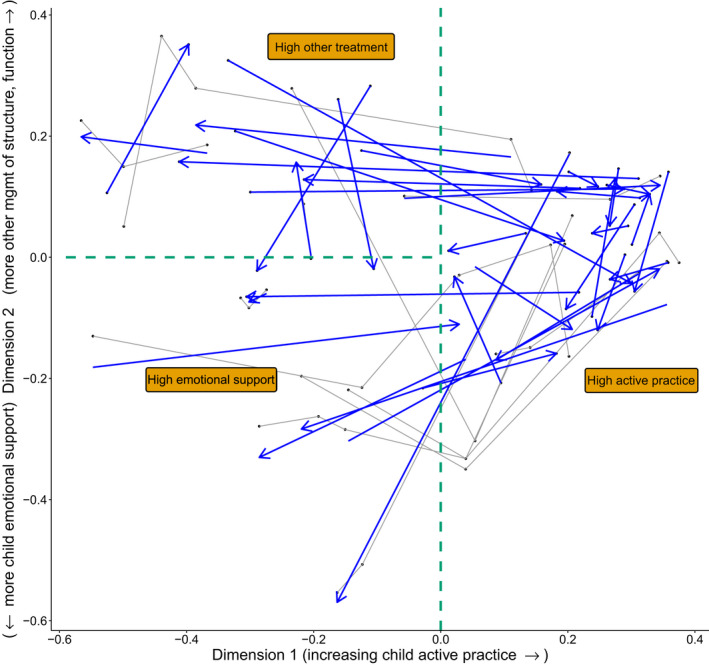
Variation in rehabilitation package content and its evolution during a rehabilitation admission. Thin grey lines show paths in the rehabilitation content space after multidimensional scaling of Paediatric Rehabilitation Ingredients Measure (PRISM) data. Blue arrows show the best‐fit straight line through each child’s trajectory: the arrows’ ends reflect the child’s earliest and last PRISM estimations. The green dashed lines divide the rehabilitation space into arbitrary domains of ‘high Active Practice’ (*x* > 0), ‘high Emotional Support’ (*x* < 0, *y* < 0) and ‘high Other Management’ (*x* < 0, *y* > 0). For the justification for labelling Dimension 1 (*x*‐axis) as ‘Child Active Practice’ and Dimension 2 (*y*‐axis) as ‘Other Management’ in the positive and ‘Child Emotional Support’ in the negative directions (see ‘Results’ and scatter plots in Figure 1)

MDS converted PRISM observations to points on a scatterplot (Figure 2). The *x* and *y* coordinates of the points on this scatterplot are, respectively, the Dimension 1 and 2 scores in Figure 1 (expressed as *z* scores around a mean of zero). Figure 1 shows that the Dimension 1 (*x*) value correlates very strongly with active practice of skills by the child (henceforth abbreviated to ‘Active Practice’: *r* = 0.977, *p* < 0.001) and Dimension 2 (*y*) correlates positively with other management of child body structure and function (henceforth ‘Other Management’; *r* = 0.518, *p* < 0.001) and Family Learning (*r* = 0.356, *p* < 0.001), and strongly negatively correlates with emotional support of the child (henceforth ‘Child Emotional Support’; *r* = –0.88, *p* < 0.001).

Conventional principal components’ analysis yields very similar results with a first component loading on Active Practice accounting for 31% of variance and a second loading negatively on Child Emotional Support and positively on Other Management and Family Learning accounting for an additional 18%. A similar structure also emerged from cluster analysis (Appendix S1).

Figure 2 shows children’s treatment as points and paths in the ‘rehabilitation content space’ and emphasizes that, for some children, rehabilitation content changes markedly during an admission. Toward the right of the plot, points converge toward the *y* = 0 line, reflecting the compositional nature of PRISM data.

### PEDI‐CAT and PRISM–PEDI‐CAT relationships

**FIGURE 3:**
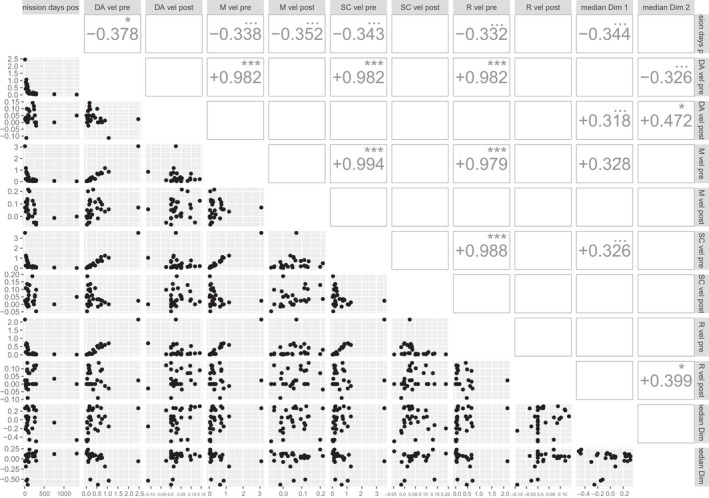
Pairwise scatterplots of pre‐ and post‐admission Pediatric Evaluation of Disability – Computer Adaptive Testing subdomain velocities, alongside median scores during admission for Paediatric Rehabilitation Ingredients Measure Dimensions 1 and 2 and interval between injury and admission. Corresponding Pearson correlations shown upper right. For clarity, correlations are only shown where *p* is < 0.1. Statistical significance symbols: •, *p* < 0.1; *, *p* < 0.05; **, *p* < 0.01; ***, *p* < 0.001. Abbreviations: DA, Daily Activity; M, Mobility; SC, Social Cognition; R, Responsibility

After deleting observations falling outside the 45‐day limits, pre‐ and post‐admission PEDI‐CAT velocities were available for 28 children (median time since injury 159 days, range 13–1384, IQR 89–219; median admission length 81 days, range 29–175; IQR 42–118). Figure 3 shows that pre‐admission PEDI‐CAT velocities were highly correlated across the four PEDI‐CAT domains (Daily Activity, Mobility, Social/Cognitive, and Responsibility) (*r* > 0.98; *p* < 0.001 in all cases), implying comparable recovery rates across these domains before admission. Figure 3 shows that some children’s post‐admission PEDI‐CAT subdomain velocities were negative (i.e. discharge scores were somewhat worse than admission scores).

Figure 3 identifies weak positive univariate correlations (although only at the *p* < 0.1 level) between median Dimension 1 (Active Practice) and pre‐admission Mobility and Social/Cognitive velocities; and between median Dimension 1 and post‐admission Daily Activity velocity. Univariate correlations between post‐admission Daily Activity and Responsibility velocities and median Dimension 2 scores reached the *p* < 0.05 threshold. More robust multivariate linear regressions were performed using the formal directed acyclic graph approach used in previous work^3^ and are summarized in Appendix S1. A statistically significant effect (standardized beta 0.44; adjusted *R* ^2^ = 0.31, *p* < 0.05) of median Dimension 1 on post‐admission PEDI‐CAT Daily Activity subdomain velocity persists after adjustment for possible pre‐admission velocity effects on rehabilitation content. The Dimension 2 rehabilitation content score was negatively correlated with pre‐admission PEDI‐CAT subdomain velocities and positively correlated with post‐admission Daily Activity and Responsibility velocities.

## DISCUSSION

To our knowledge, this is the first detailed quantitative examination of cross‐sectional and longitudinal variation in rehabilitation content after acquired brain injury, certainly in children. Three approaches – MDS, cluster analysis, and principal components analysis – yield very similar findings. Child active practice followed (with opposite signs) by child emotional support and other management of body structure and function emerge as the PRISM ingredients accounting for most of the variation in rehabilitation treatment content between and through admissions. It should be emphasized that PRISM’s conceptualization of ‘active practice’ encompasses all forms of ‘learning by doing’ irrespective of ‘target’. Depending on the clinical context, this could include practicing speaking or activities of daily living as well as many cognitive rehabilitation techniques and not just the active practice of gross and fine motor skills.

We focussed on the MDS approach here because of its explicitly spatial interpretation and a wish to explore trajectories in ‘rehabilitation content space’. The dominance of active practice is perhaps not surprising, but the opposing ‘polarities’ of Child Emotional Support and Other Management on Dimension 2 is somewhat unexpected. Further qualitative examination of what clinical populations these rehabilitation patterns are associated with will be informative. Large absolute (positive or negative) scores on Dimension 2 are associated with less active practice (*x* < 0 in Figure 2), which may reflect therapist perceptions that active practice is not needed, either because rapid recovery is happening ‘spontaneously’ or because it is thought improbable. It should also be emphasized that in scoring PRISM, raters are asked to consider what is actually being provided, which may not be everything that is needed.

In the introduction, we underlined the need to be able to quantify rehabilitation treatment (content and dose) in order to examine possible rehabilitation dose–response effects. The challenge of robust, causal inference here is underlined by the most important and novel observation of this study, namely the extent to which rehabilitation content can change during an admission. Figure 2 identifies strikingly long ‘journeys’ through the rehabilitation content space for some admissions. The length of these trajectories is a novel metric, and detailed qualitative examination of the factors driving the change in rehabilitation content in longer‐trajectory cases will be an important area of future research. In general terms, the evolution of treatment content could either reflect treatment success (early treatment goals were achieved and the team moved on to others) or failure (therapists tried something that did not appear very successful so changed approach). Our previous work^16^ suggests that therapists are generally successful in setting appropriate early rehabilitation goals, which would support the first of these interpretations.

More generally, this finding underlines the importance and magnitude of the third challenge identified in the introduction: whether therapists’ decisions to alter rehabilitation content are determinants of, or are occurring in response to, observed recovery. The findings of Table S2 support both interpretations. The negative values for Dimension 2 ‘coefficient A’ (i.e. pre‐admission PEDI‐CAT subdomain velocity is negatively correlated with median PRISM Dimension 2 score) imply that slow pre‐admission recovery is associated with less emotional support and more ‘Other Management’ rehabilitation content. However, significant positive effect sizes (standardized beta ~ 0.5, *p* < 0.05) for median Dimension 2 on post‐admission Daily Activity and Responsibility velocities persist after adjustment for this.

Figure 2 clearly demonstrates the limitations of using a median score for either PRISM dimension in these analyses. More sophisticated analytical approaches^17^ will be required to assess the effect of this time‐varying exposure. Other limitations include the necessary assumption of linearity and a minimum possible score of 20 on the day of injury in calculating pre‐admission PEDI‐CAT subdomain velocities from the admission PEDI‐CAT scores. Although we have previously shown^4^, ^6^, ^18^ that over the longer term, recovery trajectories tend to be asymptotic (fastest shortly after injury, later slowing to a plateau), here the pre‐admission velocities are simply used as a crude proxy for therapists’ semi‐quantitative perceptions of ‘recovery so far’, as we hypothesize this may inform possibly implicit judgements of ‘recovery potential’ and consequent rehabilitation content treatment choices. For this purpose we would argue the average velocity is adequate and it is in fact useful that a child admitted late after injury the average velocity may reflect that the curve has long since started to flatten; a therapist may be implicitly aware that ‘there was some early recovery but there has not been much improvement for the last several weeks’ and adjust content accordingly. Other important limitations of this work include the small sample, with the potential for both imprecise estimates due to patient heterogeneity, and under‐adjustment due to omitted confounders. All models also assumed appropriate variable distributions, with only linear relationships explored and no interactions considered (again, due to the small sample size).

We have set this work in the context of the overarching goal of being able to identify rehabilitation dose–response effects with confidence (with the ultimate aim of improving rehabilitation outcomes through comparative effectiveness research). Previous efforts have largely been in adults, in stroke,^19^, ^20^, ^21^ and traumatic brain injury.^22^, ^23^ Although these studies acknowledged that rehabilitation could not be treated as a ‘black box’,^24^, ^25^, ^26^ their approaches to ‘unpacking’ it had important limitations. For example, an essentially negative dose–response study in adult stroke^27^ approached characterization of rehabilitation content via the delivering profession (physical, occupational, speech therapy hours) and a distinction between ‘impairment‐focus’ and ‘function‐focus’. We have demonstrated that crude therapy‐hour dose measures that do not consider content can show paradoxical dose–response relationships (the ‘highest doses’ being received by those with the poorest outcomes).^3^ We have also discussed the inadequacy of using a treatment target (e.g. ‘dressing practice’) to define rehabilitation ingredients (see Forsyth et al.^9^ and particularly the online supplementary material) and prefer models based on treatment theory^26^, ^28^, ^29^ defining ingredients, mediators of effect, and target outcomes.^30^

The hoped‐for benefit of this approach to rehabilitation‐content parsing is the ability to examine rehabilitation dose–response effects. In a recently published pilot study that focussed specifically on rates of gross motor recovery,^3^ we found strong and robust correlations with the PRISM active practice fraction. The present study’s effect sizes and model adjusted *R* ^2^ values are generally smaller than the previous study,^3^ indicating greater unexplained variability, but they are still statistically significant and clinically plausible. One likely contribution to this discrepancy is the more ‘downstream’ perspective on outcome captured by the PEDI‐CAT compared to the Gross Motor Function Measure. Small negative post‐admission PEDI‐CAT velocities were seen in some children. Discussion with treating clinicians included the suggestion that discharge scores reflected subtler difficulties that may have been overlooked at admission. Similarly, PEDI‐CAT captures real world performance and thus may be affected by fatigue and motivation. Although the 61 children with PRISM data represented only two‐thirds of the admissions over the period, there was no evidence of selection bias, and some children in this sample were years post‐injury, increasing the chance that function had already largely plateaued.

Despite these complications, however, the ready demonstration of statistically significant correlations in small samples here and in our previous work^3^ contrasts with the relative lack of success in identifying similar correlations in adult studies after TBI^22^, ^27^ and supports the PRISM approach to rehabilitation content parsing. The challenge now is to interpret these correlations within a robust causal‐inference framework.

## Supporting information


Appendix S1: Supporting material.

## Data Availability

Data availability limited due to privacy/ethical restrictions. Please approach authors.
