# Supplementary material for: Rehabilitation after paediatric acquired brain injury: Longitudinal change in content and effect on recovery
Source: Dev Med Child Neurol. 2022 Mar 9;64(9):1168–75. doi: 10.1111/dmcn.15199 (PMC9544058; doi:10.1111/dmcn.15199)
Supplement: Supplementary file 1 — Appendix S1: Supporting material. [file DMCN-64-1168-s001.docx]

Appendix S1

**Dimensionality reduction of PRISM data**

*Principal Components Analysis*

Results of a standard Principal Components Analysis (PCA) of *rcomp*-transformed PRISM scores (with centering but without scaling as data is already normalised) using the *princomp* function with default settings in R. The Figure S1 biplot shows a very similar factor structure to that revealed by the Multidimensional Scaling (MDS) reported in the main paper: a first component loading strongly on Child Active Practice and a second component on Child Emotional Support and Child Other Management (with opposite signs). The cumulative variance in the PRISM data accounted for by successive principal components is shown in Table S1.

**Figure S1**

| **Component** | **1** | **2** | **3** | **4** | **5** |
| --- | --- | --- | --- | --- | --- |
| Standard deviation | 0.271 | 0.205 | 0.190 | 0.162 | 0.151 |
| Proportion of variance accounted for | 0.310 | 0.178 | 0.152 | 0.111 | 0.096 |
| Cumulative Proportion of variance accounted for | 0.31 | 0.49 | 0.64 | 0.75 | 0.85 |

*Supplementary Table 1. Importance of components of PCA*

***Result of hierarchical cluster analysis***

Results of hierarchical cluster analysis of *rcomp*-transformed PRISM scores, using a set of dissimilarities for 61 PRISM observations (drawn at random, one per child). Initially, each observation is assigned to its own cluster and then the algorithm proceeds iteratively, at each stage joining the two most similar clusters and recalculating dissimilarities until there is just a single cluster. Dissimilarity is calculated as Euclidean distance and the “single linkage” clustering method is used.

******

*Figure S2*

**Directed Acyclic Graph**

A directed acyclic graph (DAG) was developed to capture hypothesised underlying data-generating processes. This is shown in Figure S3 and is directly equivalent to that used in our previous work [3]. With the exception of assumed independence between age and gender it is saturated (i.e. every possible forward causal transmission is accommodated). Thus the DAG accommodates the possibility that injury type (coded as “hypoxic”, “traumatic” or “other ABI”) may be influenced by age and gender; that pre-admission PEDI-CAT subdomain (DA, M, SC or R) velocity may be influenced by age, gender and injury type; that “rehabilitation dose” may be influenced by age, gender, injury type and pre-admission PEDI-CAT subdomain velocity; and that post-admission PEDI-CAT subdomain velocity may be influenced by age, gender, injury type, pre-admission PEDI-CAT subdomain velocity and “rehabilitation dose”.

The “rehabilitation dose” measure was the median score on Dimension 1 (~ Active practice) or Dimension 2 (Other ↔︎ Emotional support) for the admission (as appropriate). As in previous work [3] very similar results were obtained using a rehabilitation dose measure multiplying median PRISM dimension score by a median “intensity” measure derived from the Therapy Intensity (TI) scale of the Rehabilitation Complexity Scale [27] which describes total “therapist effort” on an 1-4 ordinal scale (less than daily treatment; daily, one provider; therapist + assistant, ~25-30 hours per week; and ≥2 therapists, ≥30 hours/week) (data not shown).

*Figure S3. DAG of hypothesised data-generating processes.*

|  | PEDI-CAT subdomain | Daily Activity (DA) | Mobility (M) | Social Cognition (SC) | Responsibility (R) |
| --- | --- | --- | --- | --- | --- |
|  |  | Standardised betas | | | |
| Dimension 1 | Coefficient A  (effect of pre-admission PEDI-CAT velocity on median PRISM dimension 1 score) |  |  |  |  |
|  | Coefficient B  (effect of pre-admission PEDI-CAT velocity on post-admission PEDI-CAT velocity) | *NS* |  |  |  |
|  | Coefficient C  (effect of PRISM dimension 1 on post-admission PEDI-CAT velocity) | 0.44*  Adjusted *R^2^* 0.31 |  |  |  |
| Dimension 2 | Coefficient A  (effect of pre-admission PEDI-CAT velocity on median PRISM dimension 2 score) | -0.34*  Adjusted *R^2^* 0.39 | -0.30•  Adjusted *R^2^* 0.36 | -0.30•  Adjusted *R^2^* 0.36 | -0.30•  Adjusted *R^2^* 0.36 |
|  | Coefficient B  (effect of pre-admission PEDI-CAT velocity on post-admission PEDI-CAT velocity) | *NS* |  |  |  |
|  | Coefficient C  (effect of PRISM dimension 2 on post-admission PEDI-CAT velocity) | 0.52*  Adjusted *R^2^* 0.28 |  |  | 0.53*  Adjusted *R^2^* 0.11 |

Supplementary Table 2. Path coefficients for the size of the Path A (pre-admission velocity on PRISM component), Path B (pre-admission velocity on post-admission velocity) and Path C (independent effect of PRISM component on post-admission velocity after adjusting for pre-admission velocity) for each of Dimension 1 and Dimension 2 with the DA, M, SC and R PEDI-CAT subdomains. Figures are standardised betas. Statistical significance symbols as per Figure 1 (• = *p* < 0.1; * = *p* < 0.05; ** = *p* < 0.01; *** = *p* < 0.001). Empty cells reflect models where the adjusted *R^2^* is < 0.1.
